# Single-Photon Tracking for High-Speed Vision †

**DOI:** 10.3390/s18020323

**Published:** 2018-01-23

**Authors:** Istvan Gyongy, Neale A.W. Dutton, Robert K. Henderson

**Affiliations:** 1 School of Engineering, Institute for Integrated Micro and Nano Systems, The University of Edinburgh, Edinburgh EH9 3FF, UK; robert.henderson@ed.ac.uk; 2 STMicroelectronics, Imaging Division, Tanfield, Edinburgh EH3 5DA, UK; neale.dutton@st.com

**Keywords:** single-photon counting, single-photon avalanche diode, quanta image sensor, machine vision, optical tracking

## Abstract

Quanta Imager Sensors provide photon detections at high frame rates, with negligible read-out noise, making them ideal for high-speed optical tracking. At the basic level of bit-planes or binary maps of photon detections, objects may present limited detail. However, through motion estimation and spatial reassignment of photon detections, the objects can be reconstructed with minimal motion artefacts. We here present the first demonstration of high-speed two-dimensional (2D) tracking and reconstruction of rigid, planar objects with a Quanta Image Sensor, including a demonstration of depth-resolved tracking.

## 1. Introduction

Quanta Image Sensors (QIS), and photon counting sensors in general, are enjoying considerable research interest, thanks to ongoing developments in enabling solid-state technologies [1]. A recent breakthrough has been the demonstration of Complementary Metal-Oxide Semiconductor (CMOS) image sensors attaining deep-sub-electron read noise [2] at room temperature and without the avalanche multiplication of Single-Photon Avalanche Diode (SPAD) sensors. On the application side, algorithms have been proposed for low-light object classification and tracking based on streams of photon counts [3], as well as image reconstruction from low photon count QIS data [4]. 

The basic output of a (single-bit) QIS is a binary bit-plane, with each constituent photodetector giving a value of 0 (for no photon detected) or 1 (at least one photon detected), with negligible read noise. These binary states (“jots” in QIS terminology) are then summed in space and/or time to form a spatiotemporally oversampled greyscale image frame. The flexibility in aggregating jots represents a distinct advantage in QIS imagers, enabling image composition to be tailored to specific applications. For instance, the “cubicle” for summing jots can be adapted according to the spatial-or-temporal variability of light intensity in a scene, so as to reduce data rates while preserving detail [5]. For faint blinking objects in a scene, it is possible to carry out signal-only summation (in time) to suppress the background. Using this approach, the effective sensitivity of a QIS was increased significantly in single molecule localization microscopy [6]. QIS processing schemes have also been suggested for high-dynamic-range (HDR) imaging [7,8].

**Figure 1:**
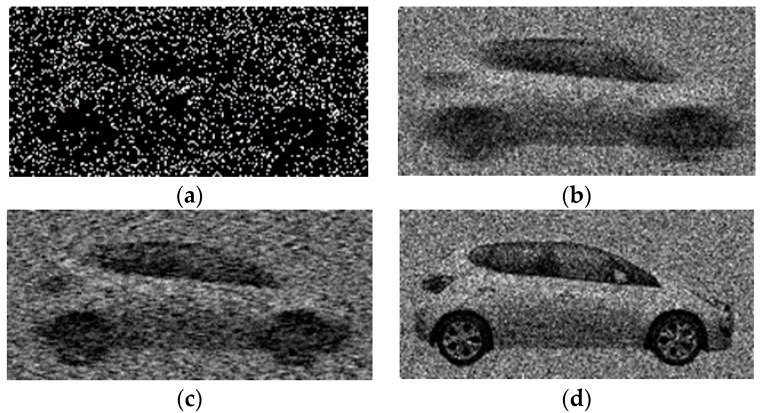
Synthetic Quanta Image Sensor (QIS) images of a car, with a maximum photon rate of 0.2 photons/pixels/bit-plane, typical of low light conditions: (**a**) Single bit-plane exposure (10 µs); (**b**) Sum of 50 bit-plane exposures of a car moving at 300 km/h; (**c**) Image b after motion deblurring using the Wiener Filter (deconvwnr function in Matlab, with the assumption of a noise-power-to-signal-power ratio of 0.05); (**d**) Sum of 50 bit-planes for static car.

SPAD implementations of QIS offer high frames rates of >100 kfps, making them ideal for tracking high-speed objects in low light. However, in a photon-starved environment there is very little detail in individual bit-planes, and summing in time to increase the bit-depth leads to motion artefacts (just as when the exposure of a conventional camera is increased). This is illustrated in Figure 1 using a set of synthetic images of a car. A single bit-plane (Figure 1a) offers limited detail, whilst summing even a modest number of bit-planes leads to significant motion blur (Figure 1b). Schemes for removing motion blur in conventional cameras typically estimate the point spread function of the distortion, either using a sequence of (blurred) images [9] or using an additional sensor (an inertial sensor or a secondary image sensor [10]). Deconvolution is then applied to the blurred images in post-processing. However, in the case of our example, even with knowledge of the exact motion of the car, deconvolution (Figure 1c) gives little visible improvement in the sharpness of the image, failing to reproduce some of the finer details seen on the image of the still car (Figure 1d). This is due to the low photon counts (and as such, the considerable shot noise) in the image to be processed. Similarly, standard motion tracking schemes based around optical flow [11,12] that perform computations on the spatiotemporal gradient of image intensity are difficult to apply in low-photon, fast-changing image sequences.

Rather than correcting composed image frames, QIS enables photon streams, as represented by the sequence of bit-planes, to be transformed (in effect, photons are re-assigned between jots) prior to summation, so as to compensate for motion and obtain increased image detail [13]. This is somewhat analogous to digital image stabilization in conventional cameras, whereby a sequence of sub-exposures is taken, of which the sharpest are selected, re-aligned, and combined (a technique which is complemented by the use of gyroscope-based optical image stabilization in many cameras [14]). However, the key difference here is that the sub-exposures (bit-planes), individually taken, do not contain sufficient information for motion tracking or frame-realignment, but may be manipulated and summed extensively without any additional noise penalty. 

This paper gives the first demonstration of high-speed object tracking and reconstruction using a QIS, based around the transformation of bit-planes. It is an expanded version of Reference [15], with additional experimental results, and a more detailed exposition of the approach. The technique requires no secondary sensors (the scheme is wholly image processing-based), and is demonstrated using a SPAD camera with back-to-back exposures, all of which are used to form the final image sequence (a high temporal aperture being important for low-light conditions). We consider the removal of motion artefacts from the whole field of view as well as tracking individual moving objects. 

## 2. Materials and Methods 

### 2.1. Motion Detection

A raw-bit plane sequence *L_i_* (*i* = 1, 2, …, *n*) will generally feature considerable photon noise, making it difficult to detect motion (or other intensity changes in the image) at the level of individual jots. We therefore use a kernel (or “cubicle”) of size {*N_x_*, *N_y_*, *N_t_*}, designed to be small enough to capture spatial and temporal variations in the image scene, to compose aggregated “test” frames *I_k_* (*k* = 1, 2, …, *n*/*N_t_*). The kernel is applied in an overlapping manner in space, and a non-overlapping in time. For the analysis that follows, we shall presume that jots have a uniform photon detection efficiency *η*, and furthermore dark counts are negligible compared with true photon detections. Assuming uniform illumination across a cubicle, and following the analysis of Reference [13], the value of each resulting pixel will be a binomial count from *M* = *N_x_* × *N_y_*× *N_t_* trials and a success probability *P_x,y,k_* = 1 − e^−*ηH*^, where *H* is the quanta exposure (or mean photon arrivals/jot during the bit-plane exposure time *τ*) corresponding to the cubicle. Thus, confidence bounds can be attached to each pixel *I_x,y,k_*, as to the “true” underlying photon arrival rate. More precisely, we calculate an (approximate) confidence interval on the estimated, underlying success probability, based on the pixel value *m* of *I_x,y,k_*, using the Agresti-Cull method [16]:

(1) $${\tilde{P}}^{\max,\min}=\tilde{P}\pm z\sqrt{\frac{\tilde{P}(1-\tilde{P})}{M+z^{2}}},\;\mathrm{where}\;\tilde{P}=\frac{m+\frac{1}{2}z^{2}}{M+z^{2}},$$

*M* is the number of jots aggregated to form the pixel and *z* = 2.58 for a 99% confidence level. We can then generate a sequence of “difference” frames *D_k_*, mapping statistically significant changes in pixel values between “test” frames *I_k_*:

(2) $$D_{x,y,k}=\{\begin{array}{c} 1\\ -1\\ 0 \end{array}\begin{array}{c} \;if\;{\tilde{P}}_{x,y,k}^{\min}>{\tilde{P}}_{x,y,k-d}^{\max}\\ \;if\;{\tilde{P}}_{x,y,k}^{\max}<{\tilde{P}}_{x,y,k-d}^{\min}\\ otherwise \end{array}.$$

where *d* is an integer constant ≥1. An alternative is to generate *D_k_* by comparing *I_k_* against a fixed reference frame, say *I*_0_, representing the background of the scene, if such a frame is available.

### 2.2. Clustering and Object Tracking

**Figure 2:**
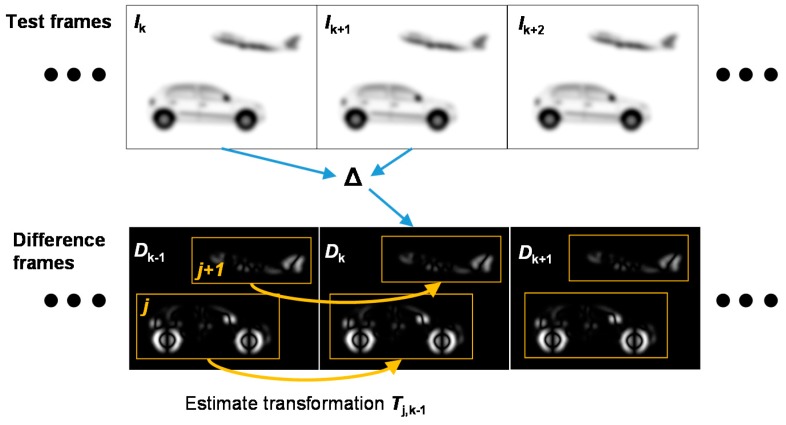
Illustration of the clustering/tracking approach: computing difference frames from the test frames give clouds of points indicating moving objects. These clouds are then clustered and bounding boxes are established. The estimated transformations between corresponding point clouds on successive difference frames give the trajectory of the relevant object.

The resulting *D_k_* frames will thus show clouds of points (pixels of value 1 or −1) corresponding to moving objects in the scene. We can separate individual objects by applying Density-Based Spatial Clustering of Applications with Noise (DBSCAN) clustering [17] to these points (using prior knowledge of the likely size of moving objects), which also identifies and filters out “outlier” points in *D_k_* resulting from photon noise. This enables regions of interest (bounding boxes, with a level of padding) to be determined around detected object motions in *D_k_*. We then take each region of interest in turn (index *j*), and, under the assumption that the detected objects may be modeled as planar objects in three-dimensional (3D) space (which can be a reasonable approximation even for non-planar objects if they are at a sufficient distance from the camera and any rotation is restricted to around the depth axis), we estimate the transformation *T_j,k_* between successive *D_k_* frames to quantify the motion (Figure 2). The transformation matrices *T_j,k_* may be computed iteratively, by optimizing a similarity metric (e.g., via a gradient descent approach, as in Matlab’s imregtform function [18]). Transformations of varying degrees of freedom may be assumed, such as rigid transformation (accounting for linear motion and rotation), similarity transformation (also including scaling, i.e., the object moving closer to/further away from the camera) or a projective transformation (if there is a change in perspective). For similarity transformation, *T_j,k_* is of the form:

(3) $$T_{j,k}=\left[\begin{array}{ccc} \lambda \cos\theta & \lambda \sin\theta & \Delta x\\ -\lambda \sin\theta & \lambda \cos\theta & \Delta y\\ 0 & 0 & 1 \end{array}\right],\;\mathrm{such}\;\mathrm{that}\;\left[\begin{array}{c} x^{\prime }\\ y^{\prime }\\ 1 \end{array}\right]=T_{j,k}\left[\begin{array}{c} x\\ y\\ 1 \end{array}\right],$$

where *λ* is the scaling factor, *θ* is the clockwise rotation, and {Δ*x*, Δ*y*} is the translation.

In the case of rigid and similarity transformations, an alternative, direct (and less computationally intensive) approach for estimating *T_j,k_* is possible (when *D_k_* is computed with respect to a fixed reference frame). We ignore the polarity of the pixel values in *D_k_*, and for each object *j*, compute the centroid {*X_j_*, *Y_j_*} of points within the corresponding bounding box. We then estimate the translation of the object by calculating the change in {*X_j_*, *Y_j_*} between successive *D_k_* frames. Scaling is obtained by calculating the mean Euclidian distance of points from {*X_j_*, *Y_j_*} (again tracking how this changes from frame to frame). For estimating rotation, we consider the spatial variance of the points in *x* and *y* (*V_x_*, *V_y_*) as, using basic trigonometry, it can be shown (for a general point cloud) that *V_x_* − *V_y_* = *C*cos(2*θ* + *φ*), where *C* and *φ* are constants and *θ* is the orientation with respect to the Cartesian axes. We note that tracking (and in particular centroiding) based on maps of changed pixel values is a very common approach in machine vision [19]. The difference here is the acquisition of photon count data (giving rise to the pixel thresholding criteria in Equation (2)), and the requirement for the full two-dimensional (2D) motion (including, for example, the orientation) of the object to be tracked for reconstruction purposes.

The object bounding boxes may be re-calculated, using DBSCAN clustering, after every *N* frames of *D_k_* (or in when a newly emerging motion is identified), between which the boxes are adapted according to the estimated motion of the enclosed object. 

### 2.3. Reconstruction of Objects and Background

For each detected object, we consider the associated sequence of transformations (interpolated in time down to the level of bit-planes), and then apply the inverse of these transformations to the original sequence of bit-planes. We then carry out aggregation in time (essentially summing bit-planes in the frame of reference of the object) so as to reconstruct the object. The result is an image frame with the reconstructed object set against a blurred background. Extracting the contour of an object from an image is a widely studied image processing problem, with [20] giving a comparison of modern techniques. Here we used the Active Contour algorithm [21], which is a popular approach for discriminating an object from its background. The algorithm does need a level of supervision to make sure results are sensible, so further work would be required to ensure the present tracking-reconstruction technique can be run in a fully automatic way.

With the motion and now the outline of the objects known, we carry out an additional sum of the bit-planes, this time untransformed but with the objects masked out, so as to recover the background (pixel values in the resulting image being scaled according to the number of frames where the pixel is object-free). Finally, we combine the enhanced images of the objects and that of the background to compose a high bit-depth image sequence, *G_i_*, at the native resolution of the camera, with minimal motion artefacts.

**Figure 3:**
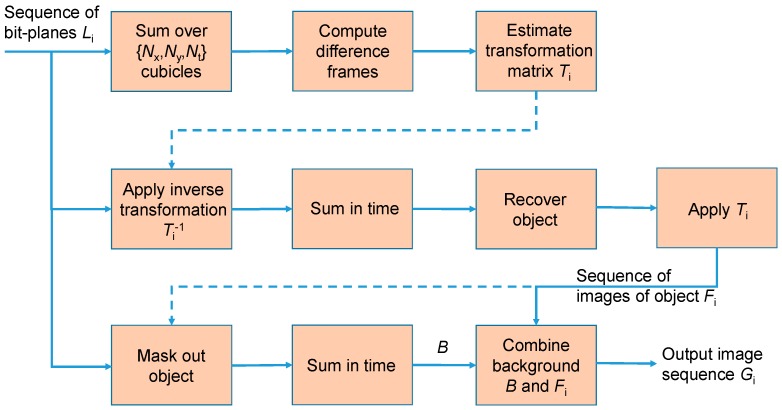
Block diagram indicating the steps in the tracking-reconstruction technique. The input is a sequence of bit-planes *L_i_* capturing a high-speed object. The scheme tracks the motion of this object (as defined by *T_i_*) and outputs a higher bit-depth image sequence, *G_i_*.

The overall objection detection-tracking-reconstruction scheme is summarized in the block diagram of Figure 3.

### 2.4. Practical Implications

In addition to the inherent requirement of a rigid, non-articulated, planar object, a number of other implicit assumptions can be identified in the above approach. There must be a sufficient contrast in light intensity between the object and the background (or some identifiable contrast within the object itself). Due to the use of difference clustering, objects should be unoccluded. Furthermore, the quality of the final reconstruction will depend on the effectiveness in extracting the contour of the object. Again, difficulties may arise if the contrast between the (motion-compensated) object and the blurred background is poor (an approximate contour may be obtainable from the difference frames in such cases). It is likely that some of these assumptions could be relaxed with more complex tracking schemes, involving dynamic probabilistic modeling, combined with some prior information on object shape and dynamics. Reference [3] gives a demonstration, using synthesized data, of tracking an inverted pendulum via a Kalman filter-based approach, adapted for sparse photon counting data.

**Figure 4:**
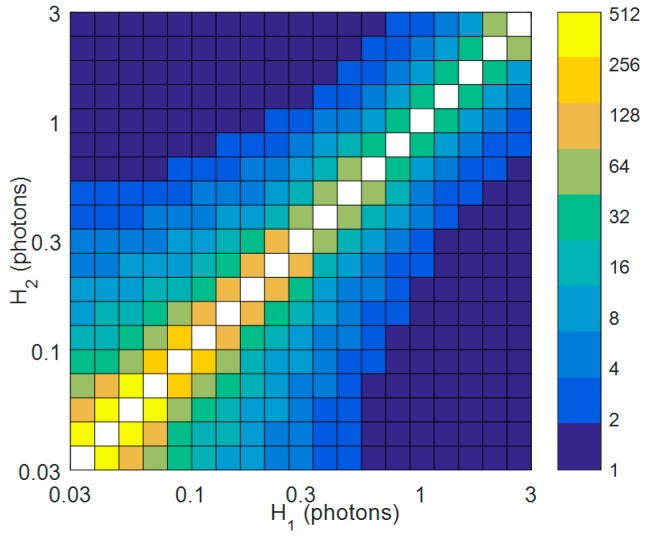
Heat map of the temporal aggregation *N_t_* required (in terms of powers of two) to detect a change of photon rate from *H*_1_ to *H*_2_ with >90% sensitivity. The photon rates are specified in terms of the mean detected photons per jot over a sub-exposure (bit-plane) and are presented on logarithmic scales. A spatial aggregation of 8 × 8 is assumed, with each data point on the heat map being obtained using Monte Carlo simulations (under the assumption of Poisson statistics) by generating 10,000 pairs of aggregated pixel realizations (from *H*_1_ and *H*_2_) at different levels of *N_t_*, and applying Equation (2).

The choice of an optimal cubicle size {*N_x_*, *N_y_*, *N_t_*} for generating the test frames is influenced by several factors. Spatially, the size of the cubicle should not exceed the size of salient features on the object, otherwise these will be averaged out, making it harder to ascertain the motion of the object. Similarly, the level of aggregation in time (*N_t_*) should be set so as to capture the motion with adequate temporal detail. At the same time, the total number of jots aggregated (*N_x_* × *N_y_* × *N_t_*) must be sufficiently large, given the level of contrast between the object and the background (or within the object), to allow pixel changes arising from the motion to be detected with high statistical certainty. This can be assessed numerically, by determining the aggregation so that a given change in the underlying photon rate H (or normalized exposure) of test frame pixels is flagged (as per Equation (2)) with a certain sensitivity (for simplicity, we assume here a photon detection efficiency of *η* = 100%). Figure 4 shows the results for a change from *H*_1_ to *H*_2_, with a range of photon rates, from 0.03 to 3 mean detected photons per jot per exposure being considered in each case. As expected, the closer *H*_1_ and *H*_2_ are, the larger the aggregation required to discriminate between them. In the case where there is prior information on the object, the results can serve as a guide for selecting *N_t_* (once the spatial aggregation *N_x_* × *N_y_* has been fixed). One can also envisage an iterative means of choosing *N_t_*, whereby a low level of temporal aggregation is first carried out, which is then progressively increased until motion is detected in the scene. 

## 3. Results

We present results from a synthetic data set designed to test the robustness of the approach, followed by experimental results obtained with a 320 × 240, 10 kfps SPAD camera [22], which is one of the highest resolution SPAD imagers available, and which also has one of the highest fill-factors at 26.8%. 

### 3.1. Simulated Data

**Figure 5:**
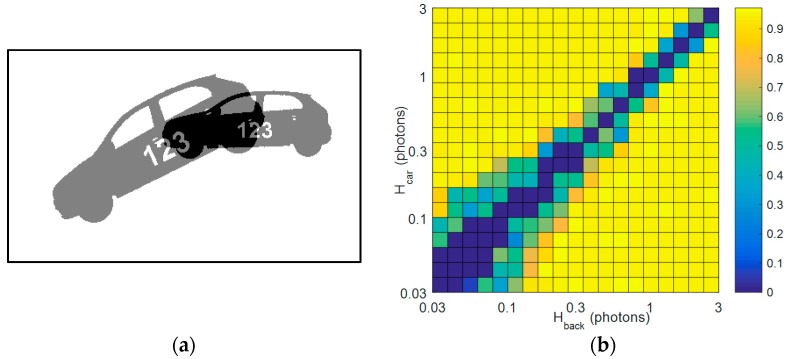
Summarized results from synthetic data set: (**a**) Two-dimensional (2D) car model (mask) used to generate data set. The car moves along *a* circular arc, with an angle to the vertical of 0.002 *i*^2^ + 0.2 *i* and a radius of *r* = 250 jots (where *i* is the index of the bit-plane), and grows in size at a rate of 0.5% per bit-plane. Shown are the initial (*i* = 1) and final (*i* = 72) positions of the car; (**b**) Heat map of the 2D correlation coefficient R between the reconstructed and still car. The coefficient R is calculated by taking the noise-free image sequence (as in panel a), and calculating the correlation between the image of the car (at *i* = 1), and the sum of the re-aligned images (*i* = 1, ..., 72), based on the trajectory extracted from the synthesized (i.e., randomized with photon noise) bit-plane sequence. Test cases where tracking was not obtained are indicated by *R* = 0. The tracking-reconstruction scheme was run with the following parameters: {*N_x_*, *N_y_*, *N_t_*} = {8, 8, 8} to create the test frames, *d* = 1 for producing the difference frames.

**Figure 6:**
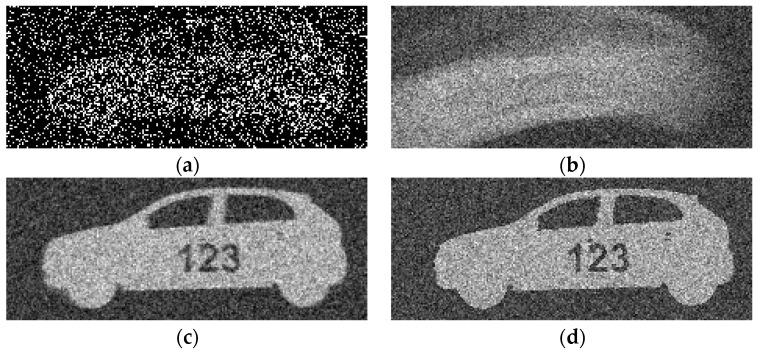
Results for synthetic data with *H_car_* = 0.35 and *H_back_* = 0.15: (**a**) Single bit-plane (*i* = 1); (**b**) Sum of bit-planes (*i* = 1, …, 72) of moving car; (**c**) Reconstructed image of moving car (*R* = 0.926); (**d**) Sum of bit-planes for still car. All images show a region of interest of 230 × 90 from the full 400 × 240 array size.

The simulated image sequences, each consisting of 72, 400 × 240 resolution bit-planes, feature a car-shaped object accelerating along a circular trajectory (Figure 5a). The speed of the car rises by 70% over the sequence, whilst the size of the car increases by 40% (growing at a rate of 0.5% per bit-plane). Assuming a bit-plane frame rate of 10 kfps, and a life-scale vehicle, the final speed of the object is several hundreds of km/h (its acceleration being unrealistically high to provide a challenging test case). The car and its background were initially assumed to have spatially uniform (and time-invariant) photon rates of *H_car_* and *H_back_*, respectively. An extensive set of image sequences were then generated to cover a range of *H_car_* and *H_back_*. Next, the tracking-reconstruction algorithm of Section 2 was applied to each sequence, and the quality of the results assessed (object transformations were estimated using the monomodal option of Matlab’s imregtform function, with the assumption of a similarity transformation, and the settings MaximumIterations = 300, MinimumStepLength = 5 × 10^−6^, MaximumStepLength = 5 × 10^−3^, RelaxationFactor = 0.9). Figure 5b plots the 2D correlation coefficient R between the reconstructed and the still car, as a function of *H_car_* and *H_back_*_._ Good correspondence is seen with the results of Figure 3, in that for combinations of *H_car_* and *H_back_* that are distinguishable according to said figure (given the cubicle size {*N_x_*, *N_y_*, *N_t_*} = {8, 8, 8} used here), good tracking is indeed obtained, leading to a reconstruction with R > 0.9. Example images are given in Figure 6 for the case of *H_car_* = 0.35 and *H_back_* = 0.15. Figure 6a shows a single bit-plane; summing the sequence of bit-planes leads to the image in Figure 6b, where the car is unrecognizable due to motion blur. Running the algorithm leads to the reconstructed image of Figure 6c, which compares well to the image of the still car (Figure 6d), with *R* = 0.943.

**Figure 7:**
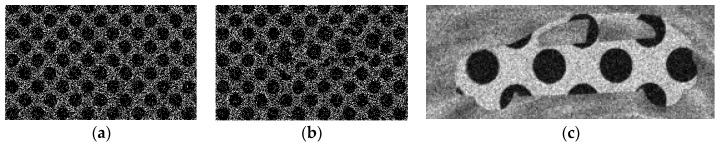
Results for synthetic data with both car and background featuring identical dotted patterns with photon rates *H*_1_ *=* 0.35 (in dot) and *H*_2_ *=* 0.05 (elsewhere). (**a**) Bit-plane at time *i* = 1; (**b**) Bit-plane at time *i* = 36; (**c**) Reconstructed vehicle (shown over a region of interest of 230 × 90). The quality of the reconstruction is *R* = 0.960.

In a subsequent data set, the uniform light intensity from the background and the body of the car were replaced by identical dotted patterns, producing a “camouflaged” car. The algorithm was run again (with the same setting as before), and was still able to reconstruct the car (Figure 7). Further testing revealed difficulties in tracking if the dots are reduced to a size approaching that of the spatial aggregation.

### 3.2. Fan and Car Sequence

**Figure 8:**
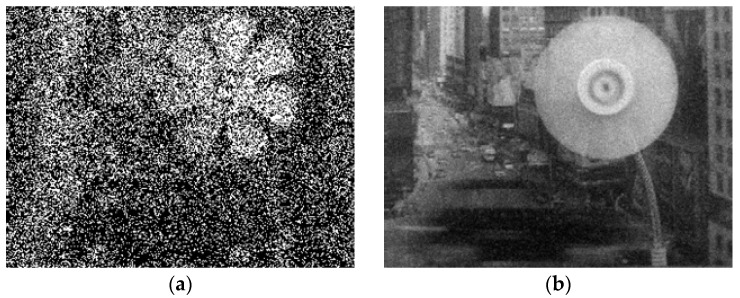

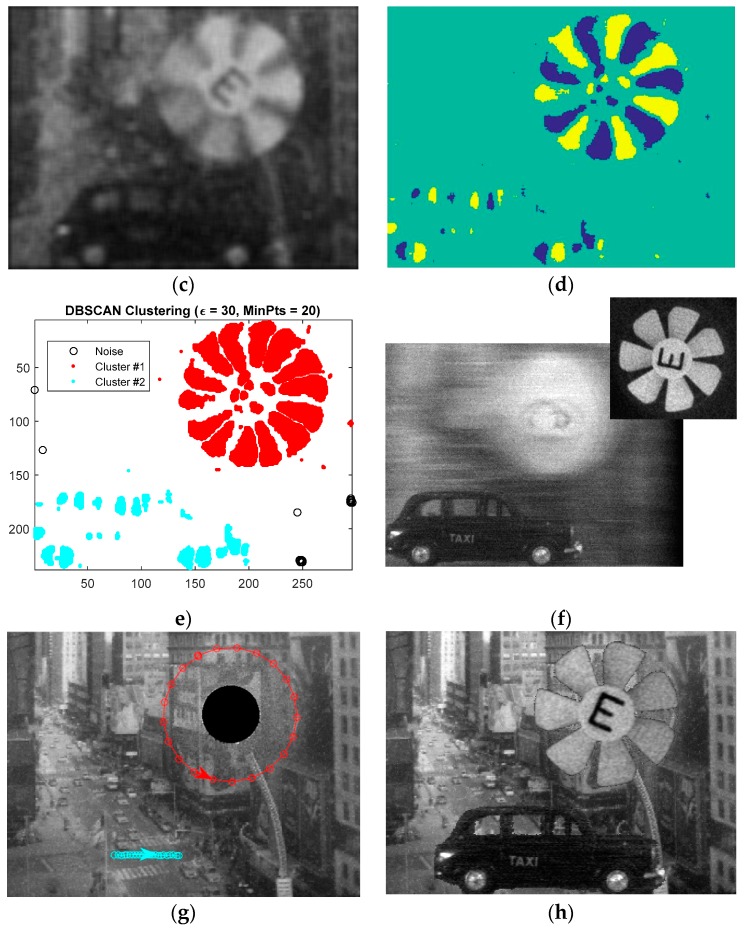
Results of fan and car test: (**a**) Single bit-plane exposure (100 μs); (**b**) Sum of *N* = 250 bit-planes (still frame from video rate image sequence, hot pixel compensation has been applied); (**c**) Test frame created by aggregating bit-planes using a kernel of size {*N_x_*, *N_y_*, *N_t_*} = {8, 8, 16}; (**d**) Difference frame computed from two test frames (*d* = 2); (**e**) Result of DBSCAN clustering using [23]; (**f**) Reconstructed images of car and fan (from *N* = 250 bit-planes); (**g**) Reconstructed background with object trajectories, over one rotation of the fan, overlaid (each division representing 16 bit-planes = 1.6 ms); (**h**) Frame from final output sequence. We note the much-improved sharpness compared with image (**b**).

To illustrate the approach in practice, we imaged a scene with a high-speed, 1:43 scale toy car, moving on a rail at 2 m/s (346 km/h full scale equivalent), together with a fan rotating at 1700 rpm. A poster was used as a backdrop. The illumination of the scene was adjusted to 10 lux to simulate reasonably dark conditions, with the camera set to take back-to-back 100 μs rolling shutter exposures to obtain a suitable signal level. An example raw bit-plane is shown in Figure 8a, showing a limited amount of detail. Although the fan can be readily identified (due to the high contrast), the car is less obvious, and no recognizable features can be seen on the poster. Aggregating bit-planes in time so as to obtain a video-rate image sequence leads to substantial motion blur (Figure 8b), such that the individual fan blades can no longer be seen, and outline of the car is also severely blurred. Standard aggregation is therefore unsuitable for obtaining detailed images of the objects and their trajectories. We instead follow the steps in the above tracking-reconstruction scheme and generate a sequence of test frames (Figure 8c), using a low level of bit-plane aggregation (we used a cubicle of size of 8 × 8 × 16 here). Another sequence of frames (difference frames) is then computed, showing significant changes in pixel values in the test frames (Figure 8d). We then apply clustering, through which the two separate motions are identified (Figure 8e). This defines appropriate bounding boxes for the difference frames, and estimating the transformations in these bounding boxes between successive frames quantifies the two motions: the linear motion of the car, and the rotation of the fan blades. We then recover a sharp image of each object in turn by applying the inverse of the associated motion to the bit-planes prior to summation. The results are given in Figure 8f, with the contours of the car and fan and the text on the car becoming visible for the first time. We extract the outlines of the objects and, based on the estimated trajectories, carry out an additional sum of the original bit-planes sequence across object-free regions only to reconstruct the background. We note that details behind the fan have become visible in the resulting image (Figure 8g). Finally, the objects are combined with the re-constructed background for an effectively blur-free image sequence (Figure 8h). 

Appendix A shows results from a similar experiment, featuring a swinging toy plane.

### 3.3. Table Tennis Ball

**Figure 9:**
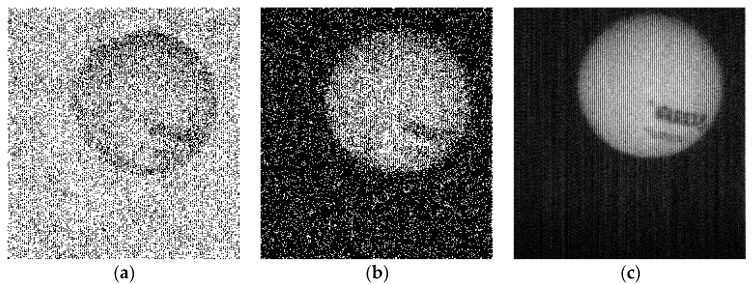
Results of time-gated imaging of fast-falling (≈10 m/s) table tennis ball, in terms of consecutive bit-plane exposures (global shutter, 100 μs), obtained using (**a**) a long time gate; (**b**) a short time gate; and (**c**) an image of the tennis ball as reconstructed from 32 short gate bit-planes through the application of the tracking scheme. A Nikon f/1.4 50 mm objective was used.

As noted above, the tracking-reconstruction scheme relies on a level of contrast in intensity between the moving objects and the background. However, in certain applications this contrast may be marginal. In such situations, we can exploit the time-resolved (or time-of-flight) capabilities of SPAD cameras, apply active illumination, and use depth (or z-position of the moving object) as a contrast agent. The SPAD camera here is ideal for fast, depth-resolved tracking, as it has dual programmable time gates which can be used in a spatially or temporally interleaved fashion. To demonstrate the approach, we imaged falling table-tennis balls, set against a white board positioned approximately 30 cm behind. The scene was illuminated with a pulsed laser source (PicoQuant 670 nm laser set to 15 MHz repetition rate) via an optical fiber, the sync signal from the laser being used to trigger the SPAD camera. Two time gates were defined, and set to alternate between odd and even bit-planes in time. The time gates (approximately 12 ns and 10 ns in width) were configured so that the longer gate would capture both the ball and the background, whilst the shorter gate would collect returning photons from the ball only. Figure 9a,b show consecutive bit-planes capturing the fast-moving tennis ball. The second bit-plane (exposed using the shorter time gate) much improved contrast, thanks to the background being suppressed through the time gating. If we consider even bit-planes only, just a short sequence is sufficient to compose (as per the approach of Section 2) a high-contrast reconstruction of the tennis ball (Figure 9c).

### 3.4. Camera Shake Compensation

Whilst the focus here is on imaging high-speed objects, a similar approach can be applied to compensate for global motion in the captured scene, for instance due to camera shake. More specifically, the procedure is as follows. As before, a sequence of test frames is created by aggregating bit-planes in time. The next step is to estimate the transformations that align these frames (we here used Matlab’s imregtform function, with the monomodal optimizer, and assumed rigid transformations). We then interpolate between the transformations (rotation and translation) associated with consecutive test frames, using cubic spline interpolation, to estimate the required alignment at the bit-plane level. Transforming the bit-planes accordingly, and aggregating for a second time, gives a motion-compensated image sequence.

**Figure 10:**
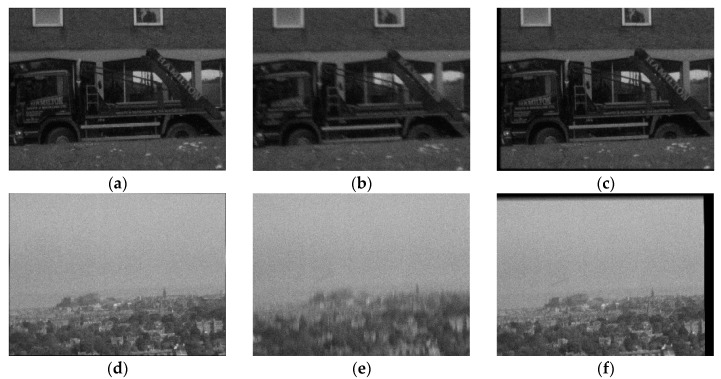
Results of camera shake test, in terms of video-rate image frames (each composed from *N* = 250 bit-planes): (**a**) truck, still camera; (**b**) truck, shaken camera (*R* = 0.79); (**c**) compensated version of image b (*R* = 0.99); (**d**) skyline, still camera; (**e**) skyline, shaken camera (*R* = 0.75); (**f**) compensated version of image of d (*R* = 0.96). *R* is the 2D correlation coefficient with respect to the image from the still camera (obtained with a tripod).

Figure 10 shows two examples of this approach, applied to the footage of a truck, and the Edinburgh skyline. In both cases, a low exposure time of 2 μs per bit-plane was used (in account of the daylight conditions), with the SPAD camera, initially placed on a tripod, and then being intentionally shaken by hand. The test frames where created by aggregating with a cubicle of {*N_x_*, *N_y_*, *N_t_*} = {1, 1, 250}, ensuring a suitably high level of bit-depth for the purposes of re-alignment (despite the long sum in time invariably leading to motion blur, realignment is still possible). The compensated images frames shown in the Figure 10 (panels (c) and (f)) are seen to be noticeably sharper than the uncompensated (test) frames (panels (b) and (e)). This is backed up by a clear increase in the indicated 2D correlation coefficients R, calculated with respect to the reference images (panels (a) and (d)), obtained with the camera still (R is normalized using the correlation of image frames in still conditions to account for the inherent frame-to-frame variability due to photon shot noise).

Further testing is planned to study the performance of the approach under different types of camera shake as well as under low light conditions.

## 4. Discussion

QIS cameras have the key benefit of providing a detailed record of individual photon detections at a high time resolution. We can exploit this information to track high-speed motion, and then re-assign photon detections spatially to compose sharp images even in potentially low light conditions. While frame re-alignment is used extensively in conventional imaging, the key difference here is that we are operating at the single-photon level.

We have here given the first demonstration of methods for extracting and compensating for motion in a QIS camera. The methods lack the sophistication of tracking schemes with dynamic probabilistic modeling (e.g., via a Kalman filter) [3], but nevertheless give an indication of the image quality improvement that can be achieved through the appropriate processing of QIS bit-planes. 

While the approach is currently implemented in software and has iterative elements, with the predicted increase in on-chip processing [24,25] in image sensors (facilitated by stacking and advanced readout schemes [26,27]), as well as the advent of advanced applications processors [28] (with computational photography and machine learning features), an integrated circuit realization is within the realm of possibility. There is thus the potential of real-time low-light vision applications, aided perhaps by active illumination and time-gated imaging for enhanced contrast, as also explored here.

## Figures and Tables

**Figure A1:**
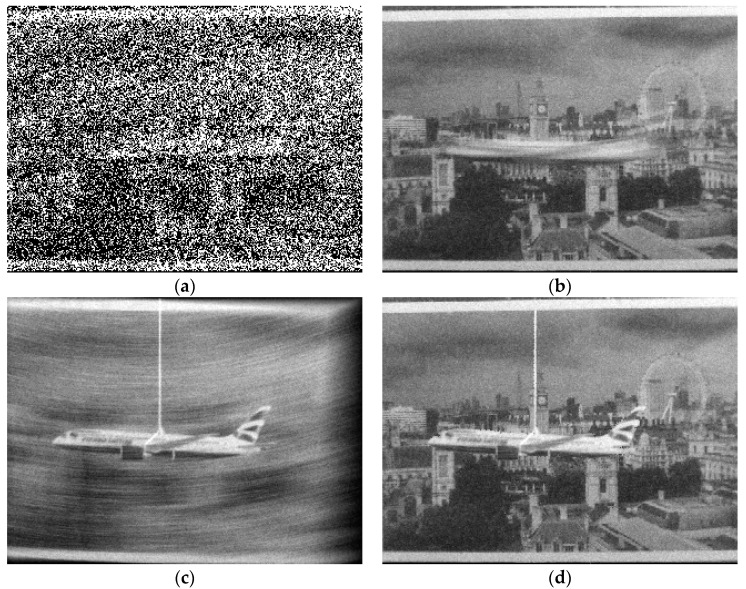
Results of plane test: (**a**) single raw bit-plane (100 μs); (**b**) sum of *N* = 250 bit-planes (still frame from video rate image sequence) (**c**) reconstructed image of plane (from *N* = 250 bit-planes); (**d**) image frame from final output sequence obtained using tracking scheme. A cubicle of {*N_x_*, *N_y_*, *N_t_*} = {8, 8, 32} was applied to create the test frames (not shown here), and difference frames were computed with *d* = 2.

## Appendix A

Figure A1 present results from an additional test, where we imaged a toy plane swinging from a cord under a light level of approximately 10 lux. Through the application of the tracking scheme, a marked improvement is seen in the sharpness of the plane (Figure A1d) as compared with simple bit-plane aggregation (Figure A1b).

## References

[1] Fossum E.R., Ma J., Masoodian S., Anzagira L., Zizza R. (2016). The quanta image sensor: Every photon counts. Sensors.

[2] Masoodian S., Ma J., Starkey D., Wang T.J., Yamashita Y., Fossum E.R. (2017). Room temperature 1040fps, 1 megapixel photon-counting image sensor with 1.1 um pixel pitch. Proc. SPIE.

[3] Chen B., Perona P. (2016). Vision without the Image. Sensors.

[4] Chan S.H., Elgendy O.A., Wang X. (2016). Images from Bits: Non-Iterative Image Reconstruction for Quanta Image Sensors. Sensors.

[5] Gyongy I., Dutton N., Parmesan L., Davies A., Saleeb R., Duncan R., Rickman C., Dalgarno P., Henderson R.K. Bit-plane processing techniques for low-light, high speed imaging with a SPAD-based QIS. Proceedings of the 2015 International Image Sensor Workshop.

[6] Gyongy I., Davies A., Dutton N.A., Duncan R.R., Rickman C., Henderson R.K., Dalgarno P.A. (2016). Smart-aggregation imaging for single molecule localisation with SPAD cameras. Sci. Rep..

[7] Fossum E.R. (2013). Modeling the performance of single-bit and multi-bit quanta image sensors. IEEE J. Electron Devices Soc..

[8] Elgendy O.A., Chan S.H. (2017). Optimal Threshold Design for Quanta Image Sensor. arXiv.

[9] Bascle B., Blake A., Zisserman A. Motion deblurring and super-resolution from an image sequence. Proceedings of the 4th ECCV ’96 European Conference on Computer Vision.

[10] Nayar S.K., Ben-Ezra M. (2004). Motion-based motion deblurring. IEEE Trans. Pattern Anal. Mach. Intell..

[11] Lucas B.D., Kanade T. An Iterative Image Registration Technique with an Application to Stereo Vision. Proceedings of the 1981 DARPA Image Understanding Workshop.

[12] Barron J.L., Fleet D.J., Beauchemin S.S. (1994). Performance of optical flow techniques. Int. J. Comput. Vis..

[13] Aull B. (2016). Geiger-Mode Avalanche Photodiode Arrays Integrated to All-Digital CMOS Circuits. Sensors.

[14] La Rosa F., Virzì M.C., Bonaccorso F., Branciforte M. Optical Image Stabilization (OIS). www.st.com/resource/en/white_paper/ois_white_paper.pdf.

[15] Gyongy I., Al Abbas T., Dutton N.A., Henderson R.K. Object Tracking and Reconstruction with a Quanta Image Sensor. Proceedings of the 2017 International Image Sensor Workshop.

[16] Agresti A., Coull B.A. (1998). Approximate is better than “exact” for interval estimation of binomial proportions. Am. Stat..

[17] Ester M., Kriegel H.P., Sander J., Xu X. A density-based algorithm for discovering clusters in large spatial databases with noise. Proceedings of the Second International Conference on Knowledge Discovery and Data Mining.

[18] Imregtform—Mathworks. https://uk.mathworks.com/help/images/ref/imregtform.html.

[19] Myler H.R. (1999). Fundamentals of Machine Vision.

[20] Arbelaez P., Maire M., Fowlkes C., Malik J. (2011). Contour detection and hierarchical image segmentation. IEEE Trans. Pattern Anal. Mach. Intell..

[21] Chan T.F., Vese L.A. (2001). Active contours without edges. IEEE Trans. Image Process..

[22] Dutton N.A., Parmesan L., Holmes A.J., Grant L.A., Henderson R.K. 320 × 240 oversampled digital single photon counting image sensor. Proceedings of the 2014 Symposium on VLSI Circuits Digest of Technical Papers.

[23] DBSCAN Algorithm-Yarpiz. http://yarpiz.com/255/ypml110-dbscan-clustering.

[24] Hseih B.C., Khawam S., Ioannis N., Muir M., Le K., Siddiqui H., Goma S., Lin R.J., Chang C.H., Liu C. A 3D Stacked Programmable Image Processing Engine in a 40 nm Logic Process with a Detector Array in a 45nm CMOS Image Sensor Technologies. Proceedings of the 2017 International Image Sensor Workshop.

[25] Nose A., Yamazaki T., Katayama H., Uehara S., Kobayashi M., Shida S., Odahara M., Takamiya K., Hisamatsu Y., Matsumoto S. A 1ms High-Speed Vision Chip with 3D-Stacked 140GOPS Column-Parallel PEs for Diverse Sensing Applications. Proceedings of the 2017 International Image Sensor Workshop.

[26] Takahashi T., Kaji Y., Tsukuda Y., Futami S., Hanzawa K., Yamauchi T., Wong P.W., Brady F., Holden P., Ayers T. A 4.1 Mpix 280fps stacked CMOS image sensor with array-parallel ADC architecture for region control. Proceedings of the 2017 Symposium on VLSI Circuits.

[27] Masoodian S., Ma J., Starkey D., Yamashita Y., Fossum E.R. A 1Mjot 1040fps 0.22 e-rms Stacked BSI Quanta Image Sensor with Cluster-Parallel Readout. Proceedings of the 2017 International Image Sensor Workshop.

[28] Inside iPhone 8: Apple’s A11 Bionic Introduces 5 New Custom Silicon Engines. http://appleinsider.com/articles/17/09/23/inside-iphone-8-apples-a11-bionic-introduces-5-new-custom-silicon-engines.

